# Official Resolutions and Messages

**Published:** 1920-05-15

**Authors:** 


### THE LATE SIR HENRY BURDETT.

### Official Resolutions and Messages.

We give below the text of the resolutions which have been carried by the following public bodies :—

### King Edward's Fund for London.

*Meeting at St. James' Palace, April 30, 1920.*

The General Council of King Edward's Hospital Fund for London desire to place on record the deep regret with which they have learned of the death of Sir Henry Burdett. Sir Henry had been associated from the very beginning with the scheme which led to the foundation of the Fund; he had been a member of the General Council from its formation in 1897 and had never missed a single meeting. He had also been closely associated with the detailed work of the Fund as a visitor for six years; a member of the Executive Committee for ten years, and of the Distribution Committee for twenty-two years. He was also closely associated with the League of Mercy, which has been the means of collecting large sums for the Fund. He was the originator of the Uniform System of Hospital Accounts, and a devoted worker in the cause of hospitals in numberless other ways. The Council are deeply sensible of the great loss which the Fund and the hospitals have sustained by his death.

### The Association of Hospital Matrons.

*Meeting at the Radcliffe Infirmary, Oxford.*

The members of the Association of Hospital Matrons present at the quarterly meeting held May 1, 1920, desire to place on record their deep regret at the loss of one who invariably placed his services at the disposal of the nursing profession, in support of the highest standard of work and the best interests of the nurses themselves.

Apart from the preceding resolutions, official intimations reached us from the following, among other bodies, which had held no meetings before this issue went to Press: The Metropolitan Hospital Sunday Fund; The League of Mercy; and The Governors of the Leicester Royal Infirmary.

Messages have also been received by the relatives from the following: The Committee of the London Hospital, conveying "their sincere sympathy in the irreparable loss . . . which the hospital world has suffered. . . . The statistics which year by year Sir Henry Burdett published on the Hospitals and Charities of the World were, in themselves, a monument of gigantic work honourable to any man; but, in addition to this, he was ever ready to advise on the smallest and insignificant matter of hospital detail, and always ready to encourage hospital officials at times of depresseion. We shall not forget that almost his last work was to champion the cause of the Voluntary System, and to fight the suggestion of State Control and State Support." The Council of the British Hospitals Association, whose honorary secretary writes: "Amongst the many varied offices which he held in hospital work, Sir Henry Burdett was Vice-President of the British Hospitals Association. As one of the prime movers in the refounding, in 1910, of the Hospitals Association under its present title of The British Hospitals Association, Sir Henry Burdett for many years took a most active part in the Association's work, and always showed the keenest interest in its general progress and development." The Incorporate Association of Hospital Officers, and The Executive Committee of the Overseas Nursing Association.

### From "The Lancet," May 8, 1920.

" . . . . If he had done no more than this he would be displayed as a valuable servant of the public, but Burdett is better known, not only to medical men but to the world, for his work in behalf of voluntary hospitals and the calling of nursing. As hospital secretary he had at his fingers' ends the real needs of these institutions and the best methods of securing voluntary assistance. Over thirty years ago he saw that there was a middle-class population, between those whose environment allowed of complete treatment in their homes and those who had a right to complete charitable assistance, for whom no institutional treatment was provided, and the result was the opening of the first hospital for paying patients in Fitzroy Square. Later he organised the first hospital conference, which led in 1884 to the institution of the Association of Hospital Managers. In these efforts, which were revolutionary in many directions in their day, he secured, now and again with some difficulty, the co-operation of the *British Medical Journal* and *The Lancet*, but later he founded his paper, *The Hospital*, more exactly to express the side of medical service with which he was personally acquainted, and to record the activities of hospital and nursing life. He had already been one of the earliest workers in the cause of the Hospital Sunday Fund, which was founded at the Mansion House in 1873 during the mayoralty of Sir Sydney Waterlow. The pattern for this great movement was supplied by the "Hospital Sunday" previously inaugurated by Canon Miller in Birmingham, and the translation of the idea to London, with its subsequent huge success, will always be associated with the names of Waterlow, Burdett, and the then editor of *The Lancet*, James Wakley.

"In 1887 Burdett established the Royal National Pension Fund for Nurses, and although in his many efforts to organise a system of training for nurses his views were often challenged, what he did for the protession of nursing was immensely valuable, as everyone will admit who knows the history of the Pension Fund. And in yet another way he has placed the philanthropic world, as well as the medical profession, under a deep obligation, for in 1883 he brought out "Hospital Charities: a Yearbook of Philanthropy," which has become the leading book of reference on all that concerns the organisation of hospitals and the life and work of their nursing staffs. It was right that such a man should receive public honour, and when Sir Henry Burdett was created K.C.B. in 1897, and K.C.V.O. some ten years later, no one who knew the devoted nature of his services thought that such rewards were over-generous.

"Personally, Burdett was an interesting and attractive man. A former colleague writes of him :—

"Those who at any time worked in close association with Sir Henry Burdett and visited him at The Lodge, Porchester Square, knew him as a kindly, hospitable, home-loving man. Until quite recent years, when death took one after another from the home circle, he was singularly happy in his family life. One always found him in the large library on the ground floor overlooking the lawn, where he spent a large part of the day and did most of his work. Even in personal conversation he never could subdue the instinct for oratorical effect, for the dramatic pause with glittering eye and wide-armed

**The late Sir Henry Burdett—(*continued*).**

gesture. His books and articles were dictated to an invisible audience, and, as a result, his literary work often had something of the diffuseness and floridity of platform speech, with the characteristic personal touch a little caricatured by cold print. Nevertheless, he was an accomplished and alert journalist, eager for the success of his papers, and well versed in the various branches of editing and publishing. His mind in general moved along large lines towards large objectives; but while he much preferred to depute the working out of details to others, he could, if need be, get down to the smallest parts of a big scheme and handle them with bold dexterity. He was a man of strong and commanding personality; by no means an 'intellectual,' but, with very great mental powers. The qualities that especially stand out in one's memory of Sir Henry Burdett are his unquenchable enthusiasm, the bigness of his ideas and projects, and his persuasive eloquence. As a colleague he was most kind, courteous, loyal and considerate; he could not be shaken from his opinions, but when he said he would do a thing he kept his word."

"To this intimate tribute we subscribe from our own knowledge. Burdett's courage, sincerity, and industry were remarkable, and the fruits of his qualities will be reaped by many to come."

**From "The British Medical Journal," May 8, 1920.**

"Thirty years ago he published the first edition of *Burdett's Hospitals and Charities*, an indispensable year-book of reference on matters relating to hospitals and other philanthropic institutions. In introducing the last edition to the public Sir Henry Burdett spoke of the happiness he had derived from 'fifty busy years in the cause of the sick, their treatment, nursing, and progressive welfare,' and added that this might be the last preface that he would address to the readers of 'Burdett.' During his Stock Exchange days, which ended in 1897, and afterwards, his eloquent appeals on behalf of charitable objects charmed many thousands of pounds out of the pockets of rich men. He did a great service to the nursing profession by organising, thirty years ago, the National Pension Fund for Trained Nurses and Hospital Officials. In this he had the active support of King Edward and Queen Alexandra (then Prince and Princess of Wales), who entertained for him a high regard, and showed it often. In the year of the Diamond Jubilee his public services were rewarded by the K.C.B., and in 1908 King Edward created him a K.C.V.O. Sir Henry Burdett was the active spirit in starting what is now the King's Hospital Fund for London; he never missed a meeting of the council from the foundation of the Fund in 1897 until last year. He had an important share also in the foundation of the League of Mercy. Again, he was the acknowledged originator of the uniform system of hospitals' accounts; others improved and extended it, but the idea was his.

"Sir Henry Burdett was a voluminous writer on hospital and nursing topics, on finance, and on various aspects of politics and public life. He was the founder and editor of The Hospital, a weekly organ of administrative medicine and institutional life, while *The Nursing Mirror*, which began as a supplement to that paper, but has long been issued separately, gained great popularity under his guidance. He paid many visits of inspection to the hospitals of the United Kingdom, and during his foreign tours he studied the work and organisation of medical charities in the United States and on the Continent. The outcome of these inquiries was his large work, *The Hospitals and Asylums of the World*, in four volumes, with a comprehensive portfolio of plans. He had an almost fatherly feeling towards medical matters and the work of the medical profession. He missed no opportunity to use his influence in the advancement of medical science and practice, and showed warm interest in post-graduate education. At the annual meeting of the British Medical Association at Brighton, in 1913, he took part by invitation in a discussion on "Hospitals in Relation to the State, the Public, and the Medical Profession" in the Section of Medical Sociology. He there expressed the view that, but for the services the hospitals were rendering, the Insurance Act would be a dead letter. He argued, too, that if only for financial reasons a whole-time salaried State medical service would never come to pass."

